# Therapeutic efficacy of chloroquine for the treatment of *Plasmodium vivax* malaria among outpatients at Hossana Health Care Centre, southern Ethiopia

**DOI:** 10.1186/s12936-015-0983-x

**Published:** 2015-11-17

**Authors:** Mesfin Assefa, Teferi Eshetu, Abdissa Biruksew

**Affiliations:** Hossana Health Care Centre, Southern Nations, Nationalities, and Peoples’ Region, Hossana, Ethiopia; Department of Medical Laboratory Sciences and Pathology, Jimma University, P.O.Box: 878, Jimma, Ethiopia

**Keywords:** *Plasmodium vivax*, Chloroquine resistance, Therapeutic efficacy, Hossana

## Abstract

**Background:**

*Plasmodium vivax* accounts for about 44 % of all malaria infection in Ethiopia. Chloroquine (CQ) is the first-line treatment for vivax malaria in Ethiopia. Chloroquine-resistant (CQR) *P. vivax* has been emerging in different parts of the world to compromise the efficacy of the drug and pose both health and economic impact in the developing world. The current study was aimed at assessing the therapeutic efficacy of CQ for the treatment of vivax malaria among outpatients at Hossana Health Care Centre, southern Ethiopia.

**Methods:**

A one-arm, 28-day follow-up, in vivo therapeutic efficacy study was conducted from 5 April to 25 June, 2014. Sixty-three patients aged between four and 59 years were enrolled with microscopically confirmed *P. vivax* infection. All patients were treated with CQ 25 mg/kg for 3 days. Recurrence of parasitaemia and clinical conditions of patients were assessed on days 1, 2, 3, 7, 14, 21, and 28 during the 28-day follow-up period. Haemoglobin (Hb) level was determined on day 0, day 28 and on day of recurrence of parasitaemia by using portable spectrophotometer.

**Results:**

Of the total 63 patients included in the study, 60 (95.2 %) completed their 28-day follow-up; three patients were excluded from the study: one patient due to vomiting of the second dose of drug, one patient due to *Plasmodium falciparum* infection and one patient lost to follow-up during the study. During enrolment, 35 (53.3 %) had a history of fever and 28 (46.7 %) had documented fever. The geometric mean of parasite density on day of enrolment was 3472 parasites/μl. Among these, two patients had recurrent parasitaemia within the 28-day follow-up. CQ was found to be efficacious in 96.7 % of the study participants except two treatment failures detected. The failure might be due to late parasitological failure among these two patients who had recurrent parasitaemia within the 28-day follow-up.

**Conclusion:**

The current study revealed that CQ showed a high rate of efficacy (96.7 %) among the study participants even though some reports from previous studies elsewhere in Ethiopia showed an increase in CQR *P. vivax*. Thus, CQR molecular markers and regular monitoring of the pattern of resistance to CQ is needed for rapid and effective control measures of possible spread of drug resistance in the study area.

**Electronic supplementary material:**

The online version of this article (doi:10.1186/s12936-015-0983-x) contains supplementary material, which is available to authorized users.

## Background

Malaria is a major public health problem and causes much suffering and premature death in tropical Africa, Asia and Latin America and occurs in 110 countries with about 40 % of the world’s population at risk [1]. Approximately 52 million people live in malaria-risk areas in Ethiopia, with mainly seasonal, unstable transmission in the highlands and longer transmission duration in lowland areas, river basins and valleys [2].

The emergence and rapid spread of resistant parasites to anti-malarial drugs, and vectors to insecticides, respectively, has worsened the problem of control [3]. Chloroquine (CQ) is the cheapest anti-malarial drug available at peripheral level (sub-health post and health post without laboratory facilities). It is used for treatment of laboratory-confirmed *Plasmodium vivax* and symptomatic malaria [4]. However, some studies revealed that the emergence of chloraquine-resistant (CQR) strains of *P. vivax* has been alarming and affected endemic countries, such as Ethiopia [5–8], Madagascar [9], Myanmar [10], and Indonesia [11]. High levels of CQR on the northern part of the islands of New Guinea and Sumatra, Indonesia have been well documented, as well as sporadic reports from other locations [12].

The World Health Organization (WHO) recommended that drug efficacy be regularly assessed. Failure to detect the emergence of anti-malarial drug resistance could lead to a drug-resistant malaria epidemic which would have major public health and economic consequences for an area, province and country [3, 13, 14]. Therefore, monitoring of drug resistance is essential for timely changes to treatment policy, which should be initiated when the treatment failure rate exceeds 10 % at the end of follow-up [13]. However, a decision to change treatment policy may be influenced by a number of additional factors, including the prevalence and geographical distribution of reported treatment failures, health service provider and/or patient dissatisfaction with the treatment, the political and economic context, and the availability of affordable alternatives to the commonly used treatment [15].

Emergence and the spread of CQR *P. vivax* strains is becoming a major public health problem in different countries, and requires regular monitoring to control its spread [1]. There are a few reports of CQR *P. vivax* in different parts of Ethiopia [6–8]. Such evidence necessitates a need for urgent assessment to obtain information in order to develop or change treatment policies [3, 13]. This study was conducted to assess the therapeutic efficacy of CQ for the treatment of vivax malaria among outpatients at Hossana Health Care Centre, southern Ethiopia.

## Methods

### Study site and population

**Fig. 1:**
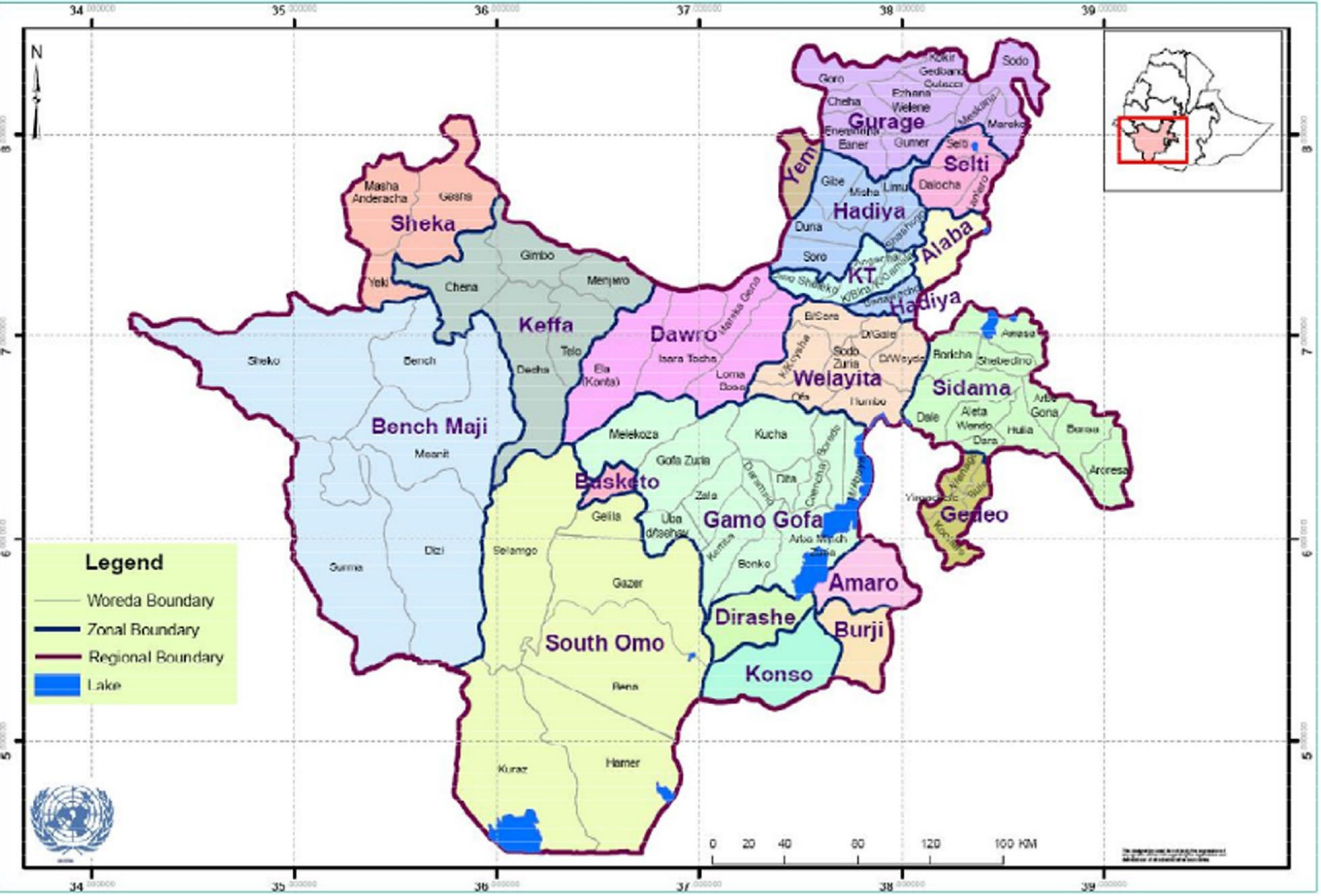
Map of the study area

The study was conducted in Hadiya Zone, Hossana Town, which is located in the Ethiopian Rift Valley (Fig. 1), in the north-western part of Southern Nation’s Nationalities and Peoples’ Region (SNNPR), 230 km south of Addis Ababa and 145 km from Hawassa. The town lies at a longitude of 10°060 N 39°590 E and latitude of 10.1°N 39.983° E. The town has a total of 104,208 inhabitants. The area has a short rainy season from March to May and a high rainfall during the main season (June–September) and is characterized by unstable and seasonal malaria, one of the main diseases in the town and its surroundings, that occurs with peak transmission following the rainy seasons [17].

The study participants were patients with confirmed *P. vivax* mono-infection on thick and thin blood film preparations and who fulfilled the inclusion criteria [13] and were seeking treatment at Hossana Health Centre during the study period. The source population were those clinically malaria-suspected individuals with fever or history of fever seeking treatment at Hossana Health Care Centre during the study period.

The inclusion criteria included age over 6 months, mono-infection with *P. vivax* detected by microscopy, asexual parasite count >250/μl, axillary temperature ≥37.5 °C or history of fever during the previous 48 h, ability to swallow oral medication, willingness to comply with the study for the duration of the study, informed consent from the patient or parent/guardian in the case of children [3, 13]. Participants who were infected with vivax malaria requiring hospitalization, or had severe malnutrition, febrile condition due to diseases other than malaria, regular medication which might interfere with anti-malarial pharmacokinetics, were pregnant and breastfeeding, were excluded from the study [3, 13].

The required sample size was calculated based on the prevalence of 3.6 % treatment failure in a study conducted in Serbo Town [7], 5 % precision, 95 % confidence level. An additional 20 % (10 patients) were expected loss to follow-up rate and withdrawal of consent during the study, and therefore 63 participants were included.

### Study design

A one-arm, prospective evaluation of clinical and parasitological responses to directly observed treatment for vivax malaria was used. Patients with *P. vivax* infection, who fulfilled the inclusion criteria were enrolled, treated and followed for 28 days. The follow-up included a fixed schedule (1, 2, 3, 7, 14, 21, and 28 days) of check-up visits and corresponding clinical and laboratory examinations [13].

Questionnaires were used to gather general information such as socio-demographic information from study participants by senior laboratory technologists and checked by the principal investigator for completeness. Clinical and laboratory information were collected as follows:

### Treatment and follow-up

A 28-day, in vivo, drug efficacy testing was done according to methods recommended by WHO [16] and the Ethiopian Ministry of Health [17]. Patients were treated with a 25 mg/kg CQ-sulfate (Addis Pharmaceuticals, Adigrat, batch number 9461, date 02/2011 and expiration 02/2016), administered for three consecutive days (10, 10 and 5 mg/kg on days 0, 1 and 2, respectively) [4, 18, 19] at Hossana Health Care Centre. All doses were administered under direct observation. Study subjects were checked for vomiting for 30 min after intake of the drug; those who vomited were retreated with the same dose. Subjects who vomited twice were excluded from the study. The study participants were advised not to take other drugs, except for patients with axillary temperature >37.5 °C who were treated with paracetamol (10 mg/kg). Patients were advised to come back to the Health Care Centre if they felt sick at any time during the follow-up period for clinical and parasitological examination. In particular, parents or guardians were instructed to bring children to the Health Care Centre at any time if they showed any sign of danger (unable to drink or breastfeed, vomiting, presenting with convulsions, lethargic or unconscious, unable to sit or stand, presenting with difficult breathing) [13].

Patients who met all the inclusion criteria were given a personal identification number and received treatment only after the study was fully explained and informed consent provided. Patients who decided to participate in the study were examined, treated and followed. Successive monitoring of parasitological and clinical responses was made on the follow-up days to each patient until day 28. The day a patient was enrolled and received the first dose of CQ was designated as day 0. Patients were informed to come for follow-up on days 1, 2, 3, 7, 14, 21, and 28. Thick and thin blood smears were prepared and examined for checking parasite clearance and/or recurrence of parasitaemia at all follow-up visits. Haemoglobin measurement was made on days 0 and 28 during follow-up and on day of recurrence of parasitaemia. Any patient who did not come to the Health Care Centre on the day of appointment was traced at his/her home and assisted by the health extension workers to complete the follow-up.

### Clinical procedures

Physical examinations such as the auxiliary temperature, weight and clinical conditions were done during the study period for all study participants.

### Laboratory procedures

Capillary blood was collected from each study participant; duplicate thick and thin blood films were made at recruitment and on each follow-up days. The blood films were stained with stained with 10 % Giemsa for 10 min, examined with100X oil immersion objective. Species identification and parasite quantification was done by trained senior medical laboratory technologist and reports were recorded on the laboratory request. The thick blood smears were used to count the numbers of asexual parasites and white blood cells (WBCs) in a limited number of microscopic fields. *Plasmodium vivax* asexual stages were counted against 200 WBCs. Parasitaemia was determined according to formula below [13].

$${\text{Parasite density }}\left( {{\text{per}}}\, \upmu {{\text{l of blood}}} \right) = \frac{{{\text{WBC }}\left( { 8000} \right) \, \times {\text{ Number of asexual parasites counted}}}}{{{\text{Number of WBC counted }}\left( { 200} \right)}}$$

Haemoglobin was measured on days 0 and 28 during follow-up for each study participants and on day of recurrence of parasitaemia. Finger-pricked blood was taken and read by portable spectrophotometer (HemoCue Hb 301 System, Sweden). Anaemia was defined according to [20] categorization.

Urine of all female participants was screened for pregnancy by Strip Test, (PR China, date 2012/11, Expiration 2014/11, Batch number W00121125.2) and those testing positive were excluded from the study.

### Study endpoints

Study participants were classified either as ‘adequate clinical and parasitological responses’, in the absence of parasite recurrence within 28 days of follow-up, or ‘treatment failure’ in case of recurrence of parasitaemia during follow-up and new infection with *Plasmodium falciparum* (within 28 days).

### Statistical analysis

Statistical Package for Social Science (SPSS) version 16 was used for data management and analysis. Data of patients having mixed infection with *P. falciparum*, lost to follow-up and vomiting were excluded from the analysis according to WHO method. The analysis included the proportion of early treatment failure (ETF), late clinical failure (LCF), late parasitological failure (LPF), and adequate clinical and parasitological response (ACPR) at day 28. Kaplan–Meier survival estimate was used to evaluate risk of therapeutic failure in study participants during follow-up period. Change in mean haemoglobin level on days 0 and 28 was compared using paired *t* test. In not normally distributed data for example age; median value was used to measure the central tendency. Parasite counts were made using geometric mean. In all analysis, p value <0.05 was considered significant.

### Ethical consideration

The study was reviewed and approved by the Ethical Review Committee of Jimma University, College of Public Health and Medical Sciences. Permission was obtained from Hadiya Zone Health Office, Hossana Town Administration Health Office and Hossana Health Centre. The purpose of the study was explained and written informed consent was obtained from each participant and parents or guardians of children.

### Data quality control

Data collectors were trained by diagnostic senior experts from the regional referral laboratory, including the principal investigator before the actual work. Competency of the data collectors was assessed and selected for the study. Standard operating procedures were strictly followed. Quality of reagents and equipment was checked each day the diagnosis of the patient sample. All *P. vivax*-positive slides on day of admission, all slides on day of recurrence and 5 % of negative slides were picked randomly from slides prepared during follow-up and were re-examined blind by experts from the regional referral laboratory.

**Fig. 2:**
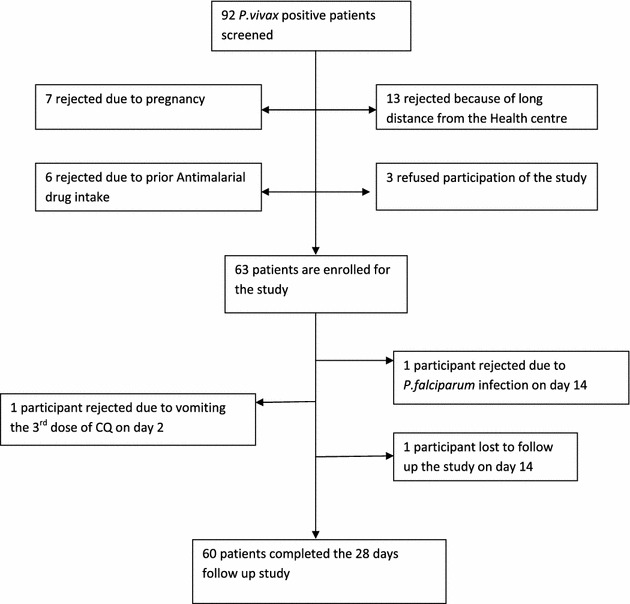
Flow chart of patients’ enrolment and follow up for CQ therapeutic efficacy study at Hossana Health Care Centre Southern Ethiopia from April to June, 2014

## Results

Of a total of 1693 patients screened for malaria at Hossana Health Care Centre, 1,412 were negative and 281 were positive for malaria. Among all positive patients, 182 were *P. falciparum*, 92 *P. vivax* and seven were mixed infection. From 92 *P. vivax*-positive patients, 63 who fulfilled the inclusion criteria were recruited. Twenty-nine patients were excluded from the study before enrolment as they did not fulfil the inclusion criteria, and of these, 13 were excluded due to long distance from the health centre, seven were pregnant, six had prior anti-malarial intake and three refused consent. From the 63 study participants, three patients were excluded from the study: one participant vomited twice during the third dose of CQ at day 2, the second participant was found infected by *P. falciparum* asexual stage on day 14, and the third patient was lost to follow-up on day 14; the remaining 60 participants completed the 28-day follow-up (Fig. 2).

**Fig. 3:**
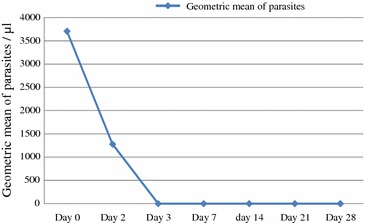
Parasite clearance following CQ treatment of *Plasmodium vivax* infected malaria patients at Hossana Health Care Centre Southern Ethiopia from April to June, 2014

Among the recruited study participants, males were higher in proportion compared to females, (35 and 25, respectively). The median age of study participants was 23 (range 4–59) (Table 1). Among the study participants, 32 (53.3 %) had a history of fever and 28 (46.7 %) had fever at the time of enrolment. The duration of illness of the patients before enrolment was 3.05 ± 1.41 (mean ± SD) days. The geometric mean of parasite at day 0 was 3472.1 parasites/μl.

**Table 1:** Socio-demographic characteristics of patients enrolled in the in vivo therapeutic efficacy study of CQ for *Plasmodium vivax* malaria at Hossana Health Care Centre Southern Ethiopia from April to June 2014

Socio-demographic variables	Total no. (%)
Age (in years)	
Median	23
Range	4–59
Gender	
Male	35 (58.3)
Female	25 (41.7)
Ethnicity	
Hadiya	40 (66.7)
Kembata	11 (18.3)
Gurage	7 (11.7)
Silte	2 (3.3)
Occupation	
Government employee	2 (3.3)
Unemployed	3 (5.0)
Student	27 (45.0)
House wife	15 (25.0)
Farmer	8 (13.8)
Daily laborer	4 (6.7)
Others	1 (1.7)
Marital status	
Single	28 (46.7)
Married	31 (51.7)
Widowed	1

Based on therapeutic failure risk calculated by the Kaplan–Meier survival analysis, the number of patients at risk on day 0 was 63, but this was attributed to only two patients with treatment failure for 28-day study. Among the 60 patients, 100 % parasite clearance was observed by day 21. However, in two patients (3.3 %) CQ treatment failure was observed during the follow-up period. This was observed in 10 and 14 years old children on days 28 and 21, respectively. This places the risk of CQ failure until day 28 at 3.3 % (Table 2).

**Table 2:** Kaplan–Meier survival estimate of risk of therapeutic failure of CQ for the treatment of *Plasmodium vivax*, at Hossana Health Care Centre Southern Ethiopia from April to June, 2014

D	N	TF	Ex	IR_D_	FCI_D_
Day 0	63	0	0	1.000	0
Day 1	63	0	0	1.000	0
Day 2	63	0	1	1.000	0
Day 3	62	0	0	1.000	0
Day 7	62	0	0	1.000	0
Day 14	62	0	2	1.000	0
Day 21	60	1	0	0.983	0.017
Day 28	59	1	0	0.967	0.033
Total		2	3		

### Parasite and fever clearance

**Fig. 4:**
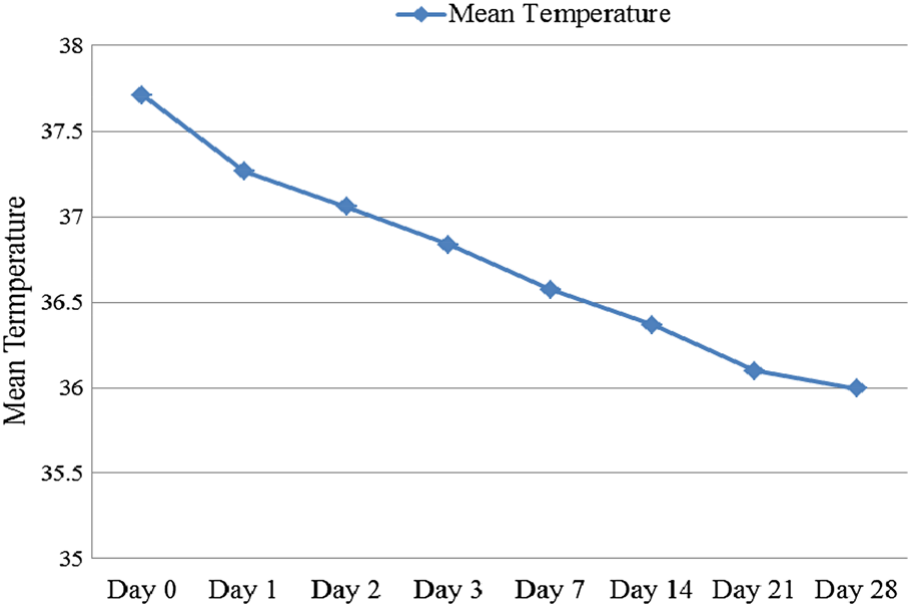
Fever clearance following CQ treatment of *Plasmodium vivax* infected malaria patients at Hossana Health Care Centre Southern Ethiopia from April to June, 2014

Parasitaemia clearance time was 72 h for all patients involved in the 28-day follow up in vivo study. At day 0 before drug administration, the geometric mean of parasitaemia of the study participants was 3708 parasites/μl of blood (maximum 10,720 parasites/μl, minimum 1600 parasites/μl). The mean body temperature (in  °C) from day of recruitment (day 0) was 37.7, 37.26, 37.0, 36.8, 36.57, 36.37, 36.10, and 35.99 °C for days 0, 1, 2, 3, 7, 14, and 28, respectively. Thus, mean body temperature of the study participants at the time of enrolment was 37.7 °C (minimum 36.5 °C, maximum 39.1 °C). Among the study participants, 32 (53.3 %) had a history of fever and 28 (46.7 %) had fever at the day of enrolment. All study participants cleared fever (mean body temperature on day 2 was 37.0 °C) following parasitaemia clearance (Figs. 3 and 4).

### Efficacy outcomes

The mean parasitaemia was 3708.15 parasites/µl of blood while the mean haemoglobin was 11.5 mg/dl on day of recruitment. Following CQ treatment, parasitaemia and fever of study participants were cleared and the mean haemoglobin concentration was improved (13.4 mg/dl). In this study, there were no early treatment failure and late clinical failures but two (3.3 %) LPFs were observed at the end of the study (on days 21 and 28). The ACPR after the 28-day follow-up was 58 (96.7 %).

**Table 3:** Recovery of haemoglobin concentration observed in *Plasmodium vivax* infected study participants following CQ treatment at Hossana Health Centre, Southern Ethiopia from April to June 2014

Hb	Age in years (D0)			Total	Age in years (D28)			Total
	1–4	5–14	>15		1–4	5–14	>15	
Mild	0	9 (30 %)	21 (70 %)	30 (50 %)	0	4 (50 %)	4 (50 %)	8 (13.3 %)
Moderate	0	1 (1.6)	0	1	0	0	0	0
Sever	0	0	0	0	0	0	0	0
Non anemia	1 (3.4 %)	6 (20.7 %)	22 (75.9 %)	29 (48.3 %)	1 (1.9 %)	12 (23.1 %)	39 (75 %)	52 (86.7 %)

### Haemoglobin recovery

On day of enrolment, 30 (50 %) patients were found to have mild anaemia: one patient was found to have moderate anaemia, no patients had severe anaemia and 29 (48.3 %) were non-anaemic (haemoglobin value ≥12 g/dl). On the other hand, a significant (p = 0.01) increase was observed in the haemoglobin level between the baseline and day 28. Among the study participants with ACPR, 52 (86.7 %) were non-anaemic and eight (13.3 %) had mild anaemia. The mean haemoglobin concentration of study participants at day of enrolment was 11.5 g/dl (ranging from 9.9 to 13.2 g/dl) and 13.4 g/dl (ranging from 10 to 14 g/dl) on day 28 (Table 3).

## Discussion

CQ has been in use both for treatment and prophylaxis in health institutions in Ethiopia. It is the anti-malarial drug recommended as first-line treatment for vivax malaria by the Federal Ministry of Health, Ethiopia [18]. However, there are some alarming reports on CQR *vivax* malaria from different malaria-endemic areas of Ethiopia [5–8]. It is becoming a major public health problem that requires rapid and effective management to control the spread of resistance, which requires proper diagnosis of cases and administration of effective anti-malarial drugs [13].

The present study showed the prevalence of falciparum and vivax malaria at Hossana Health Care Centre, southern Ethiopia. *Plasmodium falciparum*-infected patients were detected more frequently than *P. vivax* patients during enrolment of study participants. Out of 282 malaria-positive patients, 182 were *P. falciparum* patients, 92 had *P. vivax* infections, and seven had mixed infections. A report from Hossana Town health office showed that in previous reports *P. falciparum* is more prevalent than *P. vivax*, which supports this recent study.

This study, a 28-day, in vivo, therapeutic efficacy test on CQ undertaken at Hossana Health Care Centre has demonstrated two (3.3 %) LPFs. The low level of treatment failure detected in the present study was comparable with previous reports from different parts of Ethiopia. Debrezeit 2 % [5], Serbo 3.6 % [7], However, the result of this study was relatively lower when compared with high treatment failures reported from Halaba Special Woreda in southern Ethiopia, 11.7 % [8].

In the current study, two patients with CQ treatment failure were children aged 10 and 14 years, which is a similar condition to cases of treatment failure observed by studies conducted in Debrezeit and Serbo towns in Ethiopia [6, 7], in India [21] and in Indonesia [11] .

Decreased parasite density on day of recurrence was observed in this study in one patient, unlike studies done elsewhere [7, 22, 23]. Based on WHO guidelines for therapeutic efficacy study of anti-malarials, resistance is classified as ETF, LCF, LPF, or ACPR. Accordingly, this study revealed two patients with LPF, which is defined as presence of parasitaemia on any day from day 7 to 28 and axillary temperature <37.5 °C, without previously meeting any of the criteria of ETF or LCF [24, 25].

Anaemia is a major effect of malaria infection. During intra-erythrocytic development, malaria trophozoites digest haemoglobin. Thus, treatment with the appropriate drug is expected to improve patients’ haemoglobin level with time [25]. In this study, significant increase in haemoglobin concentration (p = 0.01) was observed from baseline to day 28 among patients with ACPR. In addition, one patient with treatment failure showed improvement of haemoglobin value on the day of recurrence. However, one patient with treatment failure had no haemoglobin improvement on the day of recurrence.

This study showed two (3.3 %) CQ treatment failures of vivax malaria at Hossana Health Care Centre, southern Ethiopia. However, the rate of resistance was lower compared to a previous study elsewhere in Ethiopia, which was 13.7 % [8]. It is important to caution responsible authorities to extend efforts to monitor drug resistance and treatment failure problems in all malaria-endemic parts of the country. According to WHO first-line treatment of malaria should be changed if the total failure rate exceeds 10 % [13]. It is important to survey CQ treatment failures and intervene before the level is reached that presents a public health problem.

## Conclusions

The three-dose regimen of CQ showed therapeutic efficacy (96.7 %) in the treatment of uncomplicated vivax malaria among study patients at Hossana Health Care Centre, southern Ethiopia. A 3.3 % CQ treatment failure detected in this study is LTF. Rapid clearance of fever and asexual parasitaemia was observed after CQ treatment of uncomplicated vivax malaria. Improvement in mean haemoglobin level was achieved following CQ treatment from day 0 to 28. Regular monitoring of the pattern of resistance to CQ is needed in vivax malaria-endemic areas of the country, and measures be taken rapidly and effectively to control the spread of resistance. Proper instruction and utilization of CQ should be exercised in order to avoid resistance. The prevalence of falciparum and vivax malaria observed in the study area requires strong malaria intervention measures.

## Additional file

10.1186/s12936-015-0983-x WHO haemoglobin threshold used to define anaemia in different age and gender groups [19].
