# Supplementary material for: Therapeutic efficacy of chloroquine for the treatment of Plasmodium vivax malaria among outpatients at Hossana Health Care Centre, southern Ethiopia
Source: Malar J. 2015 Nov 17;14:458. doi: 10.1186/s12936-015-0983-x (PMC4650862; doi:10.1186/s12936-015-0983-x)
Supplement: Supplementary file 1 — 10.1186/s12936-015-0983-x WHO haemoglobin threshold used to define anaemia in different age and gender groups [19]. [file 12936_2015_983_MOESM1_ESM.docx]

**Additional file 1**: WHO haemoglobin threshold used to define anaemia in different age and gender groups [[19](file:///F:\Tables.docx#_ENREF_19)].

|  | **Anaemia definition(in mg/dl)** | | | |
| --- | --- | --- | --- | --- |
| **Age or gender group** | **Non anemic** |  | | |
|  |  | **Mild Anemia** | **Moderate Anemia** | **Severe Anemia** |
| Children (0.5–5.0 years) | >11.0 | 10-10.9 | 7-9.9 | <7 |
| Children (5.0–12.0 years) | >11.5 | 11-11.4 | 8-10.9 | <8 |
| Children (12.0–15.0 years) | >12.0 | 11-11.9 | 8-10.9 | <8 |
| Women, non-pregnant (>15years) | >12.0 | 11-11.9 | 8-10.9 | <8 |
| Women, pregnant (>15)years | >11.0 | 10-10.9 | 7-9.9 | <7 |
| Male (>15years) | >13.0 | 11-12.9 | 8-10.9 | <8 |
